# Prostate transglutaminase (TGase-4) antagonizes the anti-tumour action of MDA-7/IL-24 in prostate cancer

**DOI:** 10.1186/1479-5876-9-49

**Published:** 2011-04-28

**Authors:** Richard J Ablin, Howard G Kynaston, Malcolm D Mason, Wen G Jiang

**Affiliations:** 1 Department of Pathology, University of Arizona College of Medicine, Arizona Cancer Center and BIO5 Institute, Tucson, Arizona, AZ 85724-5043 USA; 2 Metastasis and Angiogenesis Research Group, Cardiff University School of Medicine, Cardiff, UK

## Abstract

**Background:**

Transglutamiase-4 (TGase-4), also known as prostate transglutaminase, belongs to the TGase family and is uniquely expressed in the prostate gland. The functions of this interesting protein are not clearly defined. In the present study, we have investigated an unexpected link between TGase-4 and the melanoma differentiation-associated gene-7/interleukin-24 (MDA-7/IL-24), a cytokine known to regulate the growth and apoptosis of certain cancer and immune cells.

**Methods:**

Frozen sections of normal and malignant human prostate tissues and human prostate cancer (PCa) cell lines PC-3 and CA-HPV-10, cell lines expressing low and high levels of TGase-4, and recombinant MDA-7/IL-24 (rhMDA-7/IL-24) were used. Expression construct for human TGase-4 was generated using a mammalian expression vector with full length human TGase-4 isolated from normal human prostate tissues. PC-3 cells were transfected with expression construct or control plasmid. Stably transfected cells for control transfection and TGase-4 over expression were created. Similarly, expression of TGase-4 in CA-HPV-10 cells were knocked down by way of ribozyme transgenes. Single and double immunofluorescence microscopy was used for localization and co-localization of TGase-4 and MDA-7/IL-24 in PCa tissues and cells with antibodies to TGase-4; MDA-7/IL-24; IL-20alpha; IL-20beta and IL-22R. Cell-matrix adhesion, attachment and migration were by electric cell substrate impedance sensing and growth by *in vitro *cell growth assay. A panel of small molecule inhibitors, including Akt, was used to determine signal pathways involving TGase-4 and MDA-7/IL-24.

**Results:**

We initially noted that MDA-7 resulted in inhibition of cell adhesion, growth and migration of human PCa PC-3 cells which did not express TGase-4. However, after the cells over-expressed TGase-4 by way of transfection, the TGase-4 expressing cells lost their adhesion, growth and migratory inhibitory response to MDA-7. On the other hand, CA-HPV-10 cells, a cell type naturally expressing high levels of TGase-4, had a contrasting response to MDA-7 when compared with PC-3 cells. Inhibitor to Akt reversed the inhibitory effect of MDA-7, only in PC-3 control cells, but not the TGase-4 expressing PC-3 cells. In human prostate tissues, TGase-4 was found to have a good degree of co-localization with one of the MDA-7 receptor complexes, IL-20Ra.

**Conclusion:**

The presence of TGase-4 has a biological impact on a prostate cancer cell's response to MDA-7. TGase-4, via mechanism(s) yet to be identified, blocked the action of MDA-7 in prostate cancer cells. This has an important implication when considering the use of MDA-7 as a potential anticancer cytokine in prostate cancer therapies.

## Background

Transglutaminases (EC 2.3.2.13) catalyze the posttranslational modification of proteins by the formation of epsilon-(gamma-glutamyl) lysine isopeptide bonds [1]. A number of human transglutaminases (TGases), as reviewed [2] have been identified and shown to have relatively restrict distribution patterns. The intracellular forms are: tissue TGase (TGase-2), keratinocyte TGase, and hair follicle TGase; extracellular TGases include factor XIIIa (plasma TGase) and prostate TGase (TGase-4, or TGaseP). In the case of TGase-4, the focus of this study, the gene is located to 3p22-p21.33 [3] and by analysis of somatic cell hybrids, mapped to chromosome 3 [3-5]. TGase-4 has a strong pattern of distribution in the prostate [6-8].

The function of the TGase-4 is not clear. The rat homologue homologue of TGase-4 (dorsal prostate TGase or Dorsal protein 1 [DP1]) has been suggested to be responsible for the cross-linking during the copulatory plug [9] formation and may be involved in sperm cell mobility and immunogenicity to some degree [10,11]. In initial studies by others [6,7], TGase-4 expression was restricted to luminal epithelial cells. The expression pattern as observed for TGase-4 has not been found thus far for any other prostate-specific marker [6]. However, the function of this enzyme in prostate cancer is unclear. Recently, it has been shown that TGase-4 is linked to the invasiveness of prostate cancer cells [12] and participates in the regulation of the interactions between prostate cancer cells and endothelial cells, the later involving the Rock signalling pathway [13]. In addition, variants of TGase-4 have been recently reported in benign and malignant human prostate tissues [14].

As part of our continuing studies to investigate proteins interacting with TGase-4 using immunoprecipitation of proteins from the prostate gland, we identified a small panel of proteins that interacted with TGase-4, including RON (the HGF-like protein receptor) [15]. MDA-7 was one of the other proteins precipitated with TGase-4.

MDA-7 (melanoma differentiation associated gene-7), also known as IL-24, was initially identified from cancer cells and found to be up-regulated in melanoma cells [16]. Forced expression of MDA-7 in cancer cells was found to be growth inhibitory [17]. The human MDA-7 gene, mapped to 1q32.2-q41, encodes a protein with a predicted size of 23.8 kD. The secreted mature MDA-7 is a 35-40 kDa phosphorylated glycoprotein. Cell types known to express MDA-7 are diverse, including B cells, NK cells, dendritic cells, monocytes, melanocytes and melanoma cells. It is now known that MDA-7 is a differentiation-, growth-, and apoptosis-associated gene with potential utility for the gene-based therapy of diverse human cancers. The location of the MDA-7 gene is closely linked to the IL-10, IL-19, and IL-20 genes within a 195-kb region -the IL-10 family cytokine cluster. MDA-7/IL-24 functions in cells via its receptor, MDA-7R/IL-24R. The MDA-7 receptor complexes include at least the IL-20alpha and IL-20beta complex and the IL-22R and IL-20Rbeta complex. Limited information is available on the effect of MDA-7 on prostate cancer cells. Studies of adenoviral vector-induced expression of MDA-7 in human prostate cancer cells demonstrated varying degree of inhibition of growth and induction of apoptosis. It is interesting to note that Bcl-2 and Bcl-xL may differentially protect human prostate cancer cells from MDA-7 induced apoptosis [18].

In the present study, we have evaluated the biological impact of TGase-4 and MDA-7 and herein report a link between MDA-7 and TGase-4 in prostate cancer cells and tissues. In the course thereof, we have further found that the effect of MDA-7 on prostate cancer cells is dependent on the presence of TGase-4 in the cell.

## Materials and methods

### Materials and cell lines

Human prostate cancer cells, PC-3 and CA-HPV-10 were from ATCC (American Type Cell Collection, Manassas, VA, USA). Fresh frozen human prostate tissues were collected from University Hospital of Wales under the approval of the local ethical committee, obtained immediately after surgery and stored at -80°C until use.

Recombinant human MDA-7/IL-24 was purchased from R&D Systems Europe (Abingdon, Oxon, UK). Antibodies to human MDA-7/IL-24, anti-IL-20Ralpha, anti-IL-20Rbeta, and anti-IL-22R were from Santa-Cruz Biotechnologies, Inc. (Santa Cruz, CA, USA) Two antibodies to human TGase-4 were respectively purchased from Covalab (Axxora Platform, Nottingham, UK) and ABCAM (Cambridge, UK). ROCK inhibitor was from Santa-Cruz Biotechnologies, Inc. (Santa Cruz, CA, USA), Akt inhibitor, SIS3 inhibitor, PLC-gamma inhibitor, JNK inhibitor, JAK inhibitor, MET inhibitor, Wortmannin, and Wiskostatin were from Calbiochem (Nottingham, UK). Matrigel (reconstituted basement membrane) was purchased from Collaborative Research Products (Bedford, MA, USA). Transwell plates equipped with a porous insert (pore size 8 μm) were from Becton Dickinson Labware (Oxford, UK). DNA gel extraction and plasmid extraction kits were from Sigma (St. Louis, MO, USA).

### Construction of hammerhead ribozyme transgenes targeting the human TGase-4 and mammalian expression vector for human TGase-4

Hammerhead ribozymes that specifically target a GTC site of human TGase-4 (GenBank accession NM_003241), based on the secondary structure of TGase-4, were generated as previously described [12,19]. Touch-down PCR was used to generate the ribozymes with the respective primers (Table 1). This was subsequently cloned into a pEF6/V5-His vector (Invitrogen, Paisley, Scotland, UK; selection markers: ampicillin and blasticidin, for prokaryotic and mammalian cells, respectively), and amplified in *E. coli*, purified, verified and used for electroporation of prostate cancer cells. Following selection of transfected cells with blasticidin (used at 5 μg/ml) and verification, the following stably transfected cells were established: TGase-4 knock-down cells (designated here as CA-HPV-10^ΔTGase4 ^in this manuscript), plasmid only control cells (CA-HPV-10^pEFa^), and the wild type, CA-HPV-10^WT^. The CA-HPV-10^ΔTGase4 ^and the CA-HPV-10^pEFa ^cells thus created were always kept in a maintenance medium which contained 0.5 μg/ml blasticidin. A mammalian TGase-4 expression construct was prepared as previously reported [15]. PC-3 cells which express little TGase-4 were transfected with either the control vector or TGase-4 expression vector. Stably transfected cells were designated as PC-3^pEF/His ^and PC-3^TGase4exp^, for control transfection and TGase-4 expression, respectively. Pooled populations of genetically manipulated cells from multiple clones were used in the subsequent studies.

**Table 1:** Primer and oligo sequences for PCR, ribozyme and amplification of full coding sequence of prostate transglutaminase (TGase-4)

	Sense (5' -'3)	AntiSense (5' - '3)
**TGase-4 expression**	Atgatggatgcatcaaaaga	Ctacttggtgatgagaacaatcttctga

**TGase-4 (position 62)**	Atggatgcatcaaaagagc	Aggtgaaacacctgtcctc (Aactgaacctgaccgtacaaggtgaaacacctgtcctc [for Q-PCR])

**TGase-4 (position 1957)**	Ataaaatgcaccccaataaa	Ctacttggtgatgagaacaatc (Actgaacctgaccgtacacctacttggtgatgagaacaatc [for Q-PCR])

**GAPDH**	Agcttgtcatcaatggaaat	Cttcaccaccttcttgatgt

**GAPDH for Q-PCR**	Ctgagtacgtcgtggagtc	Actgaacctgaccgtacacagagatgatgacccttttg

### RNA preparation and RT-PCR

RNA from cells was extracted using an RNA extraction kit (AbGene Ltd, Surrey, UK) and the concentration quantified using a spectrophotometer (Wolf Laboratories, York, UK). cDNA was synthesised using a first strand synthesis with an oligo^dt ^primer (ABgene, Surrey, UK). PCR was performed using sets of primers (Table 1) with the following conditions: 5 min at 95°C, and then 20 sec at 94°C-25 sec at 56°C, 50 sec at 72°C for 36 cycles, and finally 72°C for 7 min. ß-actin was amplified and used as a house keeping control. PCR products were then separated on a 0.8% agarose gel, visualized under UV light, photographed using a Unisave™ camera (Wolf Laboratories, York, UK) and documented with Photoshop software.

### Quantitative analysis of TGase-4

The level of the TGase-4 transcripts in the above-prepared cDNA was also determined using a real-time quantitative PCR, based on the Amplifluor™ technology modified as previously reported [19,20]. Briefly, pairs of PCR primers were designed using the Beacon Designer™ software (version 2, Palo Alto, CA, USA), but added to one of the primers was an additional sequence, known as the Z sequence (5'actgaacctgaccgtaca'3) which is complementary to the universal Z probe (Intergen Inc., Oxford, UK). A Taqman detection kit for ß-actin was purchased from Perkin-Elmer. The reaction was carried out using the following: Hot-start Q-master mix (ABgene, Surrey, UK), 10 pmol of specific forward primer, 1 pmol reverse primer which has the Z sequence (underlined [Table 1]), 10 pmol of FAM-tagged probe, and cDNA generated from approximate 50 ng RNA. The reaction was carried out using IcyclerIQ™ (Bio-Rad, Hammel Hemstead, UK) which was equipped with an optic unit that allows real time detection of 96 reactions. The following condition was used: 94°C for 12 min, 50 cycles of 94°C for 15 sec, 55°C for 40 sec and 72°C for 20 sec. The levels of the transcripts were generated from an internal standard that was simultaneously amplified with the samples.

#### *In vitro *cell growth assay

Cells were plated into 96-well plated at 2,000 cells/well followed by a period of incubation. Cells were fixed in 10% formaldehyde on the day of plating and daily for the subsequent 5 days. 0.5% crystal violet (w/v) was used to stain cells. Following washing, the stained crystal violet was dissolved with 10% (v/v) acetic acid and the absorbance was determined at a wavelength of 540 nm using an ELx800 spectrophotometer. Absorbance represents the cell number.

#### Electric Cell-substrate Impedance Sensing (ECIS) based cell adhesion assay

Two models of ECIS instrument were used: ECIS 9600 for screening and ECIS1600R for modeling. In both systems, 8W10 arrays were used (Applied Biophysics Inc, Troy, NY, USA) [21,22]. Following treatment of the array surface with a Cysteine solution, the arrays were incubated with complete medium for 1 hr. The same number of prostate cancer cells, PC-3^pEF/His^, PC-3^TGase4exp^, or PC-3^wt ^when appropriate CA-HPV-10^ΔTGase4^, CA-HPV-10^pEF/His ^or CA-HPV-10^wt ^(300,000 per well) were added to each well. Electric changes were continuously monitored for up to 24 hr. In the 9600 system, the monitoring was at fixed 30 Hz. In the 1600R system, two conditions were recorded: 400 Hz, 4,000 Hz, 40,000 Hz for screening the nature of resistance changes and 4,000 Hz fix frequency for cell modeling. For cell adhesion and motility modeling, we employed the Rb modeling methods provided by the software of ECIS-1600R, based on a method previously reported [23]. After recording adhesion and migration at 4,000 Hz, cell behaviour was modeled using the Rb method by using a cell free well as a reference unit. Cell migration and adhesion are shown here as the resistance.

#### Immunofluorescence co-staining of TGase-4 and MDA-7 or MDA-7 receptors in cells and tissues

Frozen sections of human prostate tissues (normal and tumour) were sectioned at a thickness of 6 μm using a cryostat. The sections were mounted on super frost plus microscope slides, air dried and then fixed in a mixture of 50% acetone and 50% methanol. The sections were then placed in "Optimax" wash buffer for 5 -10 min to rehydrate. Sections were incubated for 20 min in a 10% horse serum blocking solution and probed with the primary antibodies (1:50 for anti-TGase-4, 1:100 for anti-MDA-7, anti-IL-20Ralpha, and 1:150 for anti-IL-20Rbeta and anti-IL-22R). Following extensive washings, sections were incubated for 30 min in the secondary FITC- and TRITC conjugated in the presence of HOESCHT-33258 at 10 μg/ml (Sigma, St. Louis, MO, USA). Following extensive washings, the slides were mounted using Fluorosave™ mounting media (Calbiochem, Nottingham, UK) and allowed to harden overnight in the refrigerator, before being examined. Slides were examined using an Olympus fluorescence microscope and photographed using a Hamamatsu digital camera. The images were documented using the Cellysis software (Olympus, Bristol, England, UK).

Statistical analysis was carried out using Minitab. For normality test: Anderson-Darling test and for statistical difference Student's "t" test.

## Results

### Over-expression of TGase-4 in prostate cancer cells diminishes the action of MDA-7/IL-24 in prostate cancer cells -Adhesion assays

**Figure 1:**
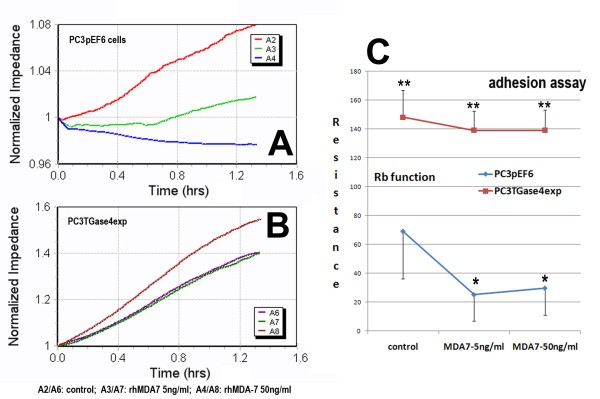
**TGase-4 expression and the cells response to rhMDA-7**. Left panel: Adhesion assay using ECIS 9600 system (A - PC-3^pEF6 ^control cells; B - PC-3^TGase4exp ^cells). Right panel (C): Cell adhesion assay using Rb modeling (ECIS 1600R, 4000 Hz). PC-3^TGase4exp ^cells showed a significant increase in cell migration when compared with the PC-3^pEF6 ^control cells (**, p < 0.01 vs the PC-3^pEF6 ^control cells). MDA-7 inhibited cell adhesion in PC-3^pEF6 cells ^(top left), a response lost in PC-3^TGase4exp ^cells. * p < 0.01 vs no MDA-7 control PC-3^pEF6 ^cells.

We first created a set of cell sublines to over-express human TGase-4(PC-3^TGase4exp^), from the prostate cancer cell line, PC-3, whose wild type had little expression of TGase-4. Using Quantitative RT-PCR analysis, PC-3^TGase4exp ^cells were found to express significantly higher levels of TGase-4 transcript (16.9 ± 2.2 copies), compared with PC-3^pEF6 ^and PC-3^wt ^(1.8 ± 0.12 for PC-3^wt ^and 2.1 ± 0.53 copies for PC-3^pEF6^, p < 0.001 vs PC-3^TGase4exp)^. The stably transfected cells were subject to testing for their adhesiveness. Figure 1 shows traces of Electric Cell-Substrate Impedance Sensing (ECIS) from an adhesion assay (A and B-left 9600 and C- right 1600R modeling). Two cell types were directly compared: PC-3 over-expressing TGase4 (PC-3^TGase4exp^) and control transfected cells (PC-3^pEF6^). In control cells (A-top left), rhMDA-7/rhIL-24 resulted in a substantial inhibition of adhesion at 50 ng/ml. PC-3^TGase4exp^, which had rapidly increased its adhesion, failed to respond to rhMDA-7 (B-left bottom). Using the 1600R and Rb based cell modeling (C-right), the same was clearly demonstrated.

### Over-expression of TGase-4 in prostate cancer cells diminishes the action of MDA-7/IL-24 in prostate cancer cells -Motility assays

**Figure 2:**
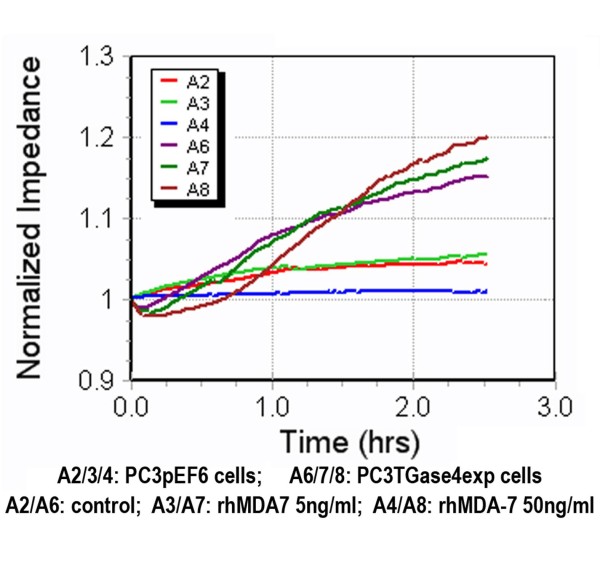
**Inhibition of cell migration by rhMDA-7 was reverted by TGase-4 expression**. PC-3^pEF6 ^control cells had a slower pace of migration in the presence of rhMDA-7. However, PC-3^TGase4exp ^cells migrated rapidly and had no response to rhMDA-7.

Here, an ECIS based wounding assay was used. Confluent monolayer cells were wounded at 6V for 30 sec which resulted in complete death of the cells over the electrode. The migration of healthy cells from the edge of the wounding to the wounding space was tracked. Similar to the changes seen with adhesion, over-expression of TGase-4 in PC-3 cells (PC-3^TGase4exp^) rendered cells, lost their response to rhMDA-7 as shown in Figure 2. PC-3 cells showed a reduced motility in the presence of rhMDA-7 (50 ng/ml), however, the response was lost in PC-3^TGase4exp^.

### A cell line naturally expressed TGase-4 responded to rhMDA7/IL-24 differently from PC-3

**Figure 3:**
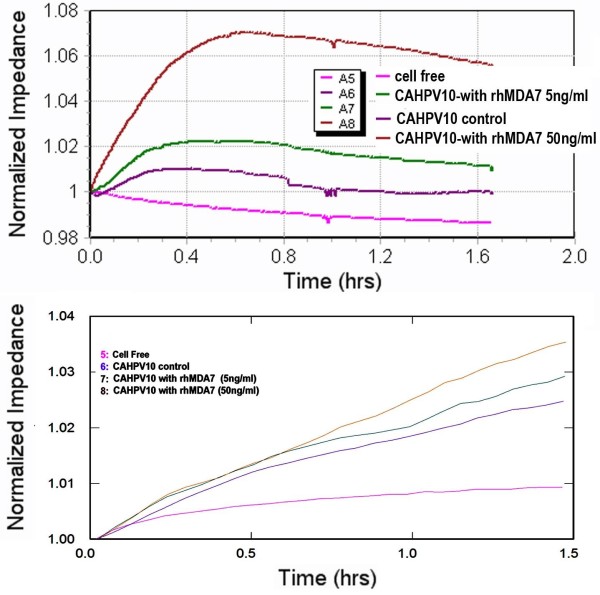
**Response to rhMDA-7 in cell adhesion (top) and migration (bottom) by CA-HPV-10 wild type cells, a cell with high levels of expression of TGase-4**.

Of all the prostate cancer cell lines in our collection, CA-HPV-10 is one that naturally expressed high levels of TGase-4 (TGase-4 transcript level in wild type being 15.8 ± 2.3 copies) [12]. We therefore tested if this cell responded differently from PC-3 cells, to the treatment of MDA-7. Unexpectedly, the CA-HPV-10 displayed, as shown in Figure 3, a very different response as evident in the two traces from 9600 (adhesion) and 1600R model (motility - wounding model). It is clear that CA-HPV-10 cells, which have high levels of TGase-4 responded to rhMDA-7 in a virtually reverse manner to PC-3, with an increased adhesion (top) and partly motility (wounding migration assay, bottom) (Figure 3).

### Effects of TGase-4 and MDA-7 on the growth of prostate cancer cells

**Figure 4:**
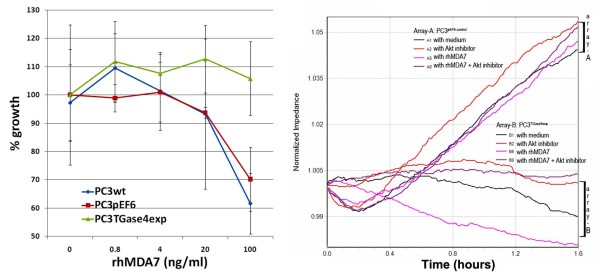
**Effects of rhMDA-7 on the *in vitro *growth of PC-3 cells (left) and the effects of the Akt inhibitor on the motility of PC-3 cells (right)**. In Array-A are PC-3^pEF6 ^cells and in Array- B are PC-3^TGase4exp ^cells. Cells were treated with or without rhMDA-7 (shown at 10 ng/ml), in the presence or absence of the Akt inhibitor (shown at 5 μM).

MDA-7 is known to have an inhibitory effect on the growth of certain cells, including some cancer cells. This was indeed seen with PC-3^wt ^and PC-3^pEF6 ^cells, as shown in Figure 4 (left). It is interesting to observe that the PC-3^TGase4exp ^cells have lost their response to rhMDA-7.

### Effects of TGase-4 expression and signalling pathways

In order to determine the potential pathways by which TGase-4 may interrupt the action of MDA-7, we used a panel of small molecule inhibitors that are either downsteam of the MDA-7 receptor pathways or known to be involved in the regulation of cell motility and growth. No significant effects were seen with the JNK inhibitor, JAK3 inhibitor, piceatannol, Wortmannin, MET inhibitor and SIS3. However, it is interesting to note that the Akt inhibitor reversed the inhibitory effects of rhMDA-7 on control PC-3 cells, but had no effect on PC-3^TGase4exp ^cells (Figure 4 right).

### Cellular co-distribution of TGase-4 and MDA-7/IL-24 in prostate cancer cells

**Figure 5:**
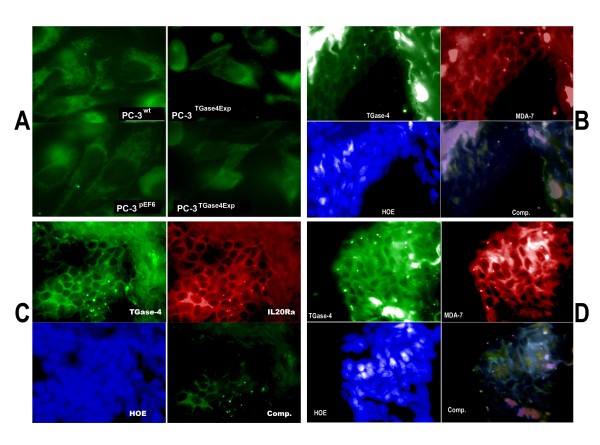
**Staining of MDA-7 in PC-3 cells (A) and co-localization of TGase-4 and MDA-7/MDA-7 receptor in human prostate tissues (B, C and D)**. A: Staining of MDA-7 in wild type (top left), control transfected cells (bottom left) and in TGase-4 expression vector transfected PC-3 cells (right two micrographs). PC-3 stained positive for MDA-7. However, after over-expressing TGase-4, staining intensity of MDA-7 is reduced. B and D: co-localization of TGase-4 and MDA-7 in normal (B) and tumour (D) prostate tissues. TGase-4 staining appears in both stroma and in the cells. C: co-localization of TGase-4 and MDA-7 receptor, IL20R, in prostate tissue. TGase-4 and IL20R have a good degree of co-localization. HOE: Nuclear staining using Hoescht 33258. Comp.: Double immunofluorescent staining reaction obtained with each of the respective antibodies in B, C and D. Magnification was ×100.

We have stained MDA-7 in prostate cancer cells. Shown in Figure 5A, PC-3 wild type cells stained for MDA-7, mostly in the cytosolic region and perinucleus areas. Over-expression of TGase-4 in the cells appeared to reduce the cytosolic staining of MDA-7 (Figure 5A).

### Tissue co-localization of TGase-4 and MDA-7/IL-24 in prostate cancer tissues

By application of double-immunofluorescent staining, we also attempted to determine if TGase-4 and MDA-7, and indeed, the MDA-7 receptor, may co-localize in normal and malignant human prostate tissues. Shown in Figure 5 (B, C and D), strong staining of TGase-4 was seen in the matrix and epithelial cells. Prostate tissues also showed staining of MDA-7 (Figure 5B and 5D) and IL-20Ra (Figure 5C). These observations demonstrated a good degree of co-localization between TGase-4, IL-20Ra and MDA-7.

## Discussion

The present study has shown that TGase-4 in human prostate cancer cells has a direct impact on the adhesive, motility and growth properties of the cell's response to rhMDA-7. Specifically, when not expressing TGase-4, cells responded well to rhDMA-7 by exhibiting a reduction of adhesion, motility and growth. However, cells expressing TGase-4 (either naturally - CA-HPV-10 or by forced expression -PC-3^TGase4exp^), had either no response to rhMDA-7 or had a marginal response opposite to those cells without TGase-4.

MDA-7/IL-24, although initially found to be up-regulated in melanoma cells [16,17], has been shown to have a growth inhibitory role in certain cancer cells [17] which include ovarian [24], colorectal [25] and glioma cancer cells [26]. The present study has shown that the MDA-7/IL-24 cytokine also inhibits the adhesion, motility and growth of prostate cancer cells. These observations place MDA-7/IL-24 within the context of a limited number of cytokines that inhibit the adhesiveness, growth and migration of cancer cells.

The most intriguing finding of the present study was that the function of MDA-7 in prostate cancer cells appears to be dependent upon the presence of TGase-4. Using two cell models, i.e., the TGase-4 expressing CA-HPV-10 and TGase-4 non-expressing PC-3 cells, we have shown that when TGase-4 is not present, MDA-7 inhibits the migration of the cells (i.e., PC-3 wild type and control cells). When TGase-4 is expressed (in CA-HPV-10 and PC-3^TGase4exp^), cells no longer respond to MDA-7.

The mechanism(s) by which TGase-4 affects MDA-7 is not clear. MDA-7/IL-24 acts via its receptor -MDA-7R/IL-24R. Receptor complexes include at least the IL-20alpha and IL-20beta complex and the IL-22R and IL-20Rbeta complex. Intracellular signalling pathways downstream of these receptors are not clear. MAPK pathways and the Fas-FasL pathway [26] have been implicated.

The present study has shown that blocking the Akt pathway using an Akt inhibitor abolishes MDA-7 induced inhibition of migration, thus indicating that Akt may be a potential pathway downstream of MDA-7. It is interesting to note that PC-3 cells over-expressing TGase-4 did not respond to MDA-7 nor the Akt inhibitor. Furthermore, inhibitors to pathways including the PLC-γ, JAK, PKC pathway, and WASP pathways, have no obvious impact on the action of MDA-7. Together, this may suggest that TGase-4 interferes with the action of MDA-7 at a stage before receptor activation. From the immunofluorescent staining of TGase-4 and MDA-7 receptor, it is clear that there is a good degree of co-localization between the TGase-4 and IL-20Ra. A possibility thus exists that TGase-4 may interact with IL-20Rs masking the site for MDA-7 to interact. More work is required to clarify the interaction of this possibility.

MDA-7 has been tested for its clinical application as an anti-cancer treatment option. Using an adenoviral-based delivery method, MDA-7 has been shown to have an anti-tumour effect in ovarian, lung, and hepatoma cancer models. MDA-7 has also been shown to increase the efficiency bevacizumab and Herceptin. Information on the effect of MDA-7 on prostate cancer cells is rather limited. However, it has been demonstrated that expression of MDA-7 in prostate cancer cells inhibits growth and induction of apoptosis [18]. Albeit, at an early stage, observations from the present study are interesting and have important clinical implications, e.g., therapeutic consideration of the use of MDA-7 would be dependent on the degree of expression of TGase-4. MDA-7 may be more sensitive in tumours that express low levels of TGase-4 and *vice versa*. This is an interesting point to consider in future pre-clinical and clinical studies.

## Conclusion

This study reports for the first time that the presence of TGase-4, a prostate specific TGase-4, has an overriding effect on a cells response to MDA-7, a potential anti-cancer cytokine. TGase-4, via mechanism(s) yet to be identified, blocked the action of MDA-7 in prostate cancer cells. This has an important implication when considering the use of MDA-7 in prostate cancer therapies.

## Competing interests

The authors declare that they have no competing interests.

## Authors' contributions

RJA and WGJ contributed to the study design, experimental work, and manuscript preparation. MDM and HGK contributed to sample collection and manuscript preparation. All of the authors read and approved the final manuscript.
